# Berberine Inhibits Human Melanoma A375.S2 Cell Migration and Invasion via Affecting the FAK, uPA, and NF-κB Signaling Pathways and Inhibits PLX4032 Resistant A375.S2 Cell Migration In Vitro

**DOI:** 10.3390/molecules23082019

**Published:** 2018-08-13

**Authors:** Jia-Fang Liu, Kuang Chi Lai, Shu-Fen Peng, Pornsuda Maraming, Yi-Ping Huang, An-Cheng Huang, Fu-Shin Chueh, Wen-Wen Huang, Jing-Gung Chung

**Affiliations:** 1 Department of Biological Science and Technology, China Medical University, Taichung 40402, Taiwan; f9512940@yahoo.com.tw (J.-F.L.); t20811@mail.cmuh.org.tw (S.-F.P.); 2 Department of Medical Laboratory Science and Biotechnology, College of Medicine and Life Science, Chung Hwa University of Medical Technology, Tainan 71703, Taiwan; kuangchi_lai@hotmail.com; 3 Department of Surgery, China Medical University Beigang Hospital, Beigang, Yunlin 65152, Taiwan; 4 Department of Medical Research, China Medical University Hospital, Taichung 40402, Taiwan; 5 Biomedical Sciences Program, Graduate School, Khon Kaen University, Khon Kaen 40002, Thailand; pornsudamaraming@gmail.com; 6 Department of Physiology, College of Medicine, China Medical University, Taichung 40402, Taiwan; yphuang@mail.cmu.edu.tw; 7 Department of Nursing, St. Mary’s Junior College of Medicine, Nursing and Management, Yilan 26644, Taiwan; haj@smc.edu.tw; 8 Department of Food Nutrition and Health Biotechnology, Asia University, Taichung 41354, Taiwan; fushin@asia.edu.tw; 9 Department of Biotechnology, Asia University, Taichung 41354, Taiwan

**Keywords:** berberine, human melanoma A375.S2, PLX4032, migration, invasion

## Abstract

Many studies have demonstrated that berberine inhibited the cell migration and invasion in human cancer cell lines. However, the exact molecular mechanism of berberine inhibiting the cell migration and invasion of human melanoma A375.S2 and A375.S2/PLX (PLX4032 induced resistant A375.S2) skin cancer cells remains unknown. In this study, we investigated the anti-metastasis mechanisms of berberine in human melanoma cancer A375.S2 cells and A375.S2/PLX resistant cells in vitro. Berberine at low concentrations (0, 1, 1.5 and 2 μM) induced cell morphological changes and reduced the viable cell number and inhibited the mobility, migration, and invasion of A375.S2 cells that were assayed by wound healing and transwell filter. The gelatin zymography assay showed that berberine slightly inhibited MMP-9 activity in A375.S2 cells. Results from western blotting indicated that berberine inhibited the expression of MMP-1, MMP-13, E-cadherin, N-cadherin, RhoA, ROCK1, SOS-1, GRB2, Ras, p-ERK1/2, p-c-Jun, p-FAK, p-AKT, NF-κB, and uPA after 24 h of treatment, but increased the PKC and PI3K in A375.S2 cells. PLX4032 is an inhibitor of the BRAFV600E mutation and used for the treatment of cancer cells harboring activated BRAF mutations. Berberine decrease cell number and inhibited the cell mobility in the resistant A375.S2 (A375.S2/PLX, PLX4032 generated resistant A375.S2 cells). Based on these observations, we suggest that the potential of berberine as an anti-metastatic agent in melanoma that deserves to be investigated in more detail, including in vivo studies in future.

## 1. Introduction

Skin cancer, including melanoma and non-melanoma skin cancer, is one of the most common types of malignancy in the white population and the incidence rate is increasing worldwide [1]. Melanoma, the most lethal skin cancer [2], is much more common in the white population than in other ethnic groups [3] and more frequent in males than in females; after age 75, the incidence of melanoma in males was almost three times that in females [4,5]. In the USA, melanoma is the fifth and seventh most common cancer among men and women, respectively [6]. Moreover, the metastatic melanoma has been recognized to be a highly aggressive malignancy and its morbidity has increased in the past years [7]. Current therapies for metastatic melanoma include chemotherapy and a variety of immunotherapeutic choices [7], however, these are still unsatisfactory. Therefore, numerous studies have focused on finding and identify new compounds from natural products for the treatment of melanoma.

Metastasis, the most important characteristic of malignant tumors, has been playing a critical role in the treatment efficacy and quality of life of patients with metastatic tumors [8]. Numerous pieces of evidence have shown that tumor cells can undergo migration, adhesion, and invasion via the lymphatic system and/or the bloodstream and they can undergo infiltration through the extracellular matrix (ECM) in order to form new tumors in other sites of the human body [9,10]. Tumor metastasis is a critical cause of cancer-related deaths [11] and is difficult to treat. ECM acts as a mechanical barrier to cell movement, thus, the degradation of the ECM is a vital step in the metastatic process [12]. Numerous pieces of evidence have shown that the matrix metalloproteinases (MMPs) degrade the ECM when facilitating a tumor invasion [13,14]. Inhibition of tumor metastasis will significantly increase the survival rate of cancer patients. Reports have shown that elevated levels of MMPs in melanoma were involved with the rapid progression of metastatic melanoma [15,16]. Thus, one of the treatments for metastasis melanoma could be blocking cancer cell migration and invasion.

Berberine, an isoquinoline alkaloid, can be isolated from the roots and bark of plants from the *Berberis* genus (Berberidaceae family) and other medical plants [17]. Berberines have biological activities such as anti-microbial [18], anti-inflammatory [19], antioxidant [20,21], and anti-cancer activities [22,23]. Numerous studies have shown that berberine decreased the cell number of many human cancer cell lines through the induction of the cell cycle arrest and apoptotic cell death [22,23,24,25]. Berberine inhibited the migration and invasion of human chondrosarcoma cells via the downregulation of the *α*v*β*3 integrin through the protein kinase C (PKC δ), c-Src, and AP-1 [26]. Berberine suppressed the migration and invasion of prostate cancer cells through the suppression of epithelial–mesenchymal transition (EMT)-related genes [27] and inhibited the invasion and metastasis of colorectal cancer cells via the down-regulation of the COX-2/PGE2- JAK2/STAT3 signaling pathway [28]. Recently, it was reported that berberine induced the apoptosis of cells and inhibited the migration of skin squamous cell carcinoma A431 cells [29]. Based on these findings, it can be asserted that berberine suppressed the migration and invasion of cancer cells through multiple mechanisms on different tumor cell types. Thus, we investigated the molecular mechanism involved in cell metastasis in human melanoma cells in vitro and the results indicated that berberine suppressed the migration and invasion of A375.S2 cells in vitro through the FAK, uPA and NF-κB signaling pathways.

## 2. Results

### 2.1. Berberine Induces Cell Morphological Changes and Decreases the Total Viability of A375.S2 Cells

**Figure 1:**
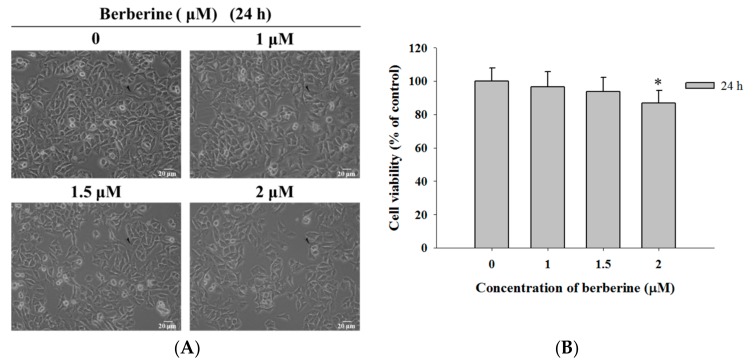
The berberine induced cell morphological changes and decreased the cell viability of A375.S2 cells. The cells (1 × 10^5^ cells/well) were incubated with berberine at different concentrations (0, 1, 1.5, and 2 μM) for 24 h. The cells were examined and photographed for morphological changes (**A**) or they were collected for the total percentage of the total viable cells (**B**) as described in Materials and Methods. * *p* < 0.05, significant difference between berberine-treated groups and the control as analyzed by one-way ANOVA analysis of variance.

As indicated in Figure 1A,B, berberine, at a 1–1.5 μM treatment dosage, did not show significant cytotoxic activity for morphology changes and reduced the total viable cell number in A375.S2 cells at 24 h (0 μM: 100% ± 7.99; 1 μM: 96.45% ± 9.07; 1.5 μM: 93.91% ± 8.22; 2 μM: 86.74% ± 7.58). However, berberine at 2 μM showed slightly induced cell morphological changes and reduced the cell number (reduced the total viable cells by 13.26%).

### 2.2. Berberine Inhibits Cell Mobility in A375.S2 Cells

**Figure 2:**
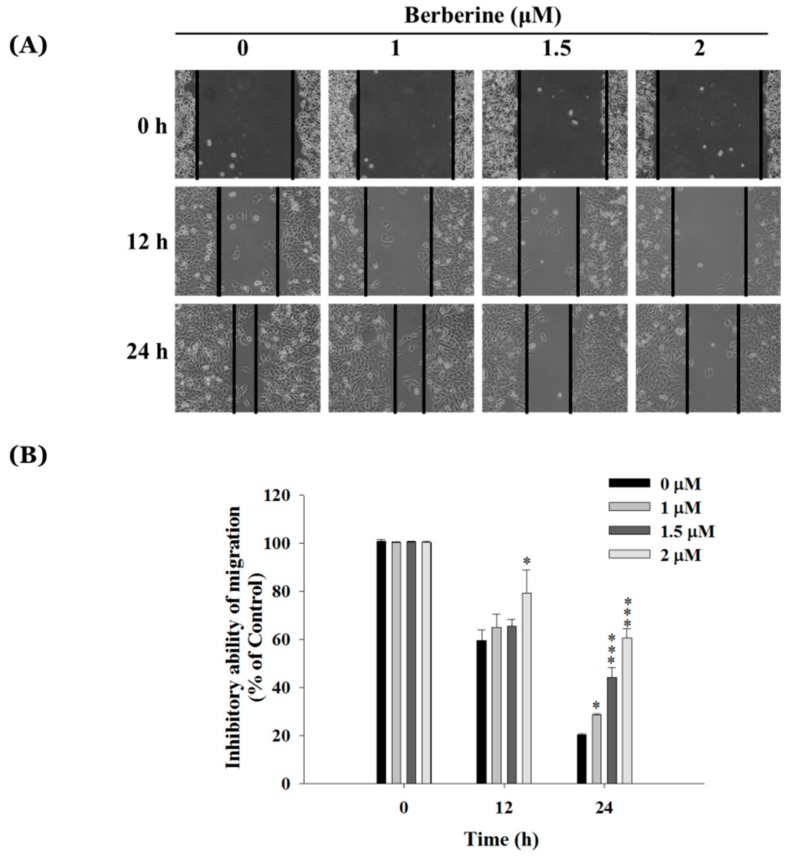
The berberine-affected in vitro wound closure of A375.S2 cells. The cells (2 × 10^5^ cells/well) were kept in 12-well plates for 24 h, scratched (wounded), and then incubated with different berberine concentrations (0, 1, 1.5, and 2 μM) for 12 and 24 h. The relative wound closures were photographed using phase contrast microscopy (**A**) and the percentage of the inhibitory ability of migration was calculated (**B**) as described in Materials and Methods. * *p* < 0.05, *** *p* < 0.001, significant difference between berberine-treated groups and the control as analyzed by one-way ANOVA analysis of variance.

The results from the wound healing assay that were presented in Figure 2A,B showed that berberine treatment at 1–2 μM inhibited the closure rate of the scratch in A375.S2 cells. The berberine treated cells remained creviced on the scratched plate but the control (untreated cells) wounds healed after 24 h of treatment. The edge distance was significantly higher in the high dosage (2 μM) group after 24 h, compared to that observed at a low dose (1 μM) (Figure 2B).

### 2.3. Berberine Affects the Matrix Metalloproteinase Activity and Cell Migration and Invasion in A375.S2 Cells

**Figure 3:**
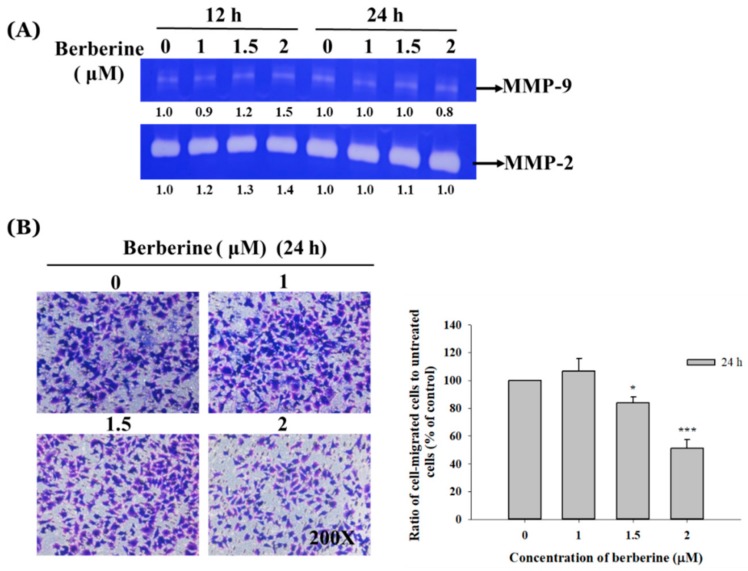

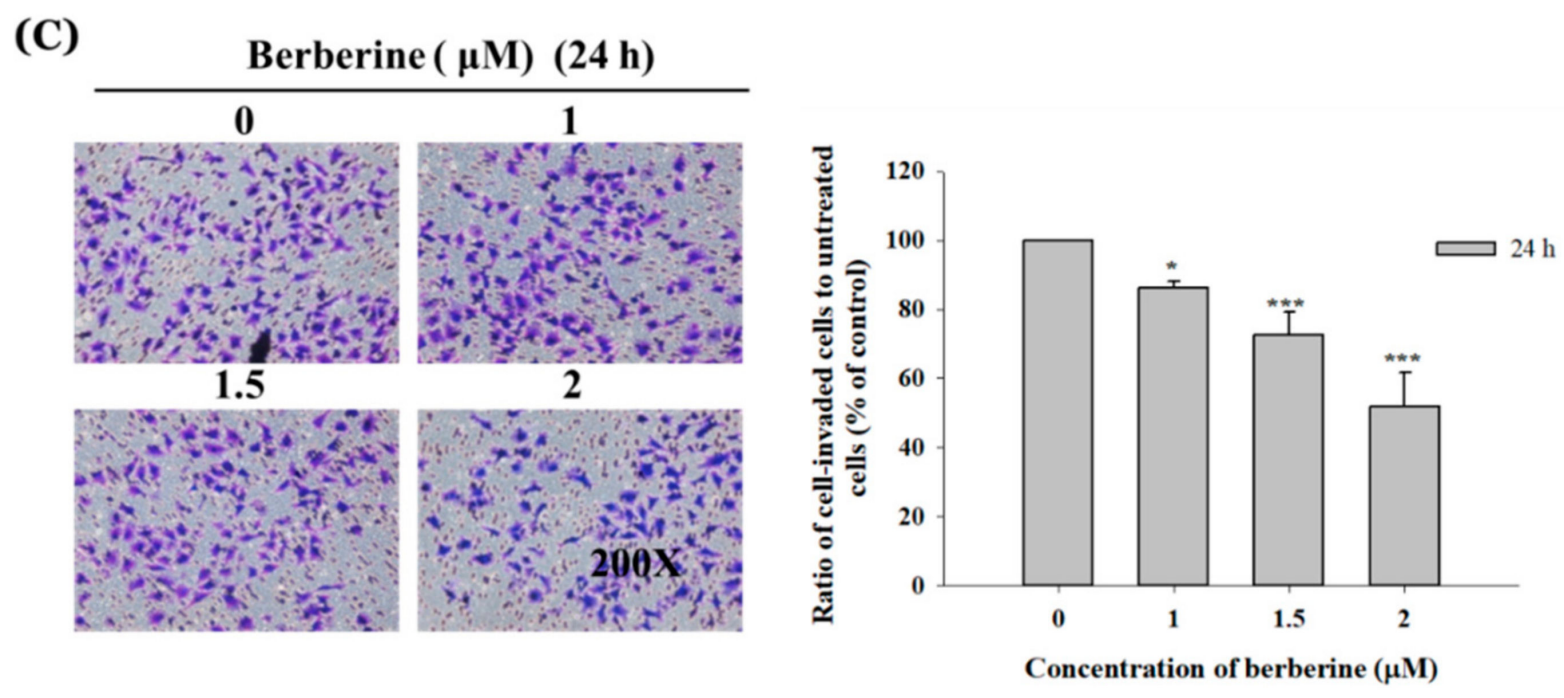
The berberine inhibited the matrix metalloproteinase (MMP) activity and suppressed the migration and invasion of A375.S2 cells in vitro. The cells (1 × 10^5^ cells/well) were incubated in 12-well plates and treated with different berberine concentrations (0, 1, 1.5, and 2 μM) for 12 and 24 h. Then the conditioned mediums were harvested for gelatin zymography assay (**A**) as described in Materials and Methods. The cells (5 × 10^4^ cells/well) were placed on transwell inserts coated with collagen for migration or with Matrigel for invasion and were treated with different berberine concentrations (0, 1, 1.5, and 2 μM) for 24 h. The A375.S2 cells penetrated to the lower surface of the transwell membrane for migration (**B**) or invasion (**C**) stained with crystal violet and photographed under a light microscope at 200×. The penetrated cells were counted as described in Materials and Method. The results were obtained from the three independent experiments. * *p* < 0.05, *** *p* < 0.001, significant difference between berberine-treated groups and the control as analyzed by one-way ANOVA analysis of variance.

After the A375.S2 cells were treated with berberine (1–2 μM) for 12 and 24 h, conditioned media were collected for determining the MMP-2 or MMP-9 activity by using gelatin zymography and the results are shown in Figure 3A. The results indicated that the berberine treatment at 1 μM concentration for 12 h and 2 μM for 24 h slightly inhibited the MMP-9 activity. The transwell chambers were coated with collagen for cell migration examination and coated with Matrigel for cell invasion examinations. The results are shown in Figure 3B,C. Figure 3B indicates that berberine (1.5–2 μM) significantly inhibited the migration of A375.S2 cells and Figure 3C indicates that berberine (1–2 μM) significantly inhibited the invasion of A375.S2 cells and that these effects are dose-dependent (Figure 3C).

### 2.4. Berberine Affects Key Metastasis-Related Proteins in A375.S2 Cells

**Figure 4:**
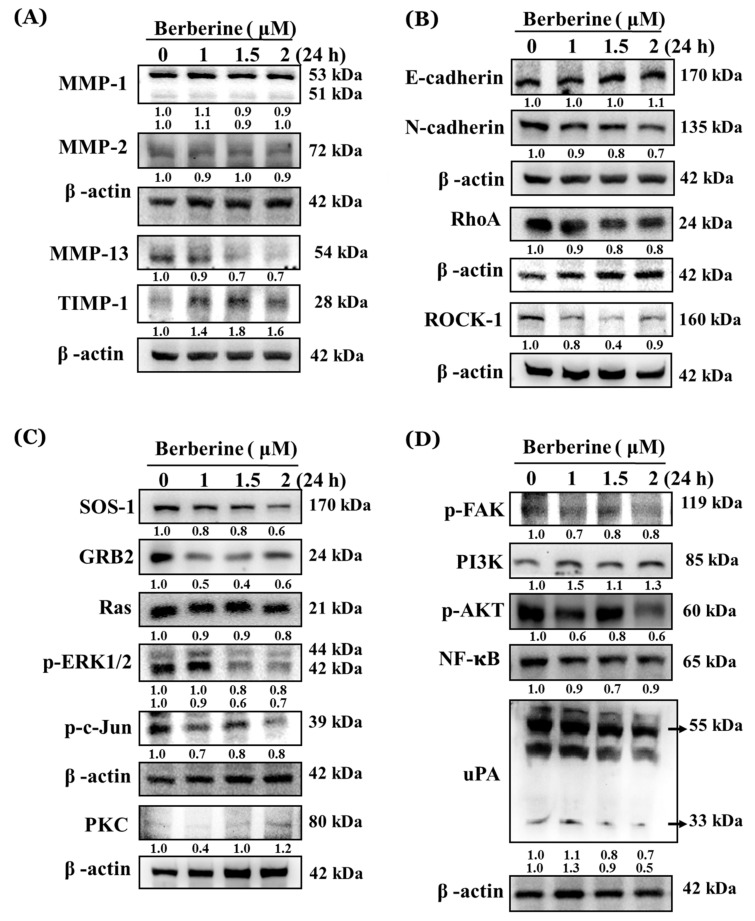
Berberine affected the levels of the associated proteins in the migration and invasion of A375.S2 cells. The cells (1 × 10^6^ cells/dish) were treated with berberine (0, 1, 1.5, and 2 μM) for 24 h. The cells were collected and the total protein was determined for sodium dodecyl sulfate polyacrylamide gel electrophoresis (SDS page) as described in the Materials and Methods. The levels of MMP-1, MMP-2, MMP-13, and TIMP-1 (**A**); E-cadherin, N-cadherin, RhoA, and ROCK1 (**B**); SOS-1, GRB2, Ras, p-ERK1/2, p-c-Jun, and PKC (**C**); p-FAK, PI3K, p-AKT, NF-κB, and uPA (**D**) expressions were estimated by western blotting as described in the Materials and Methods.

As indicated in Figure 4A–D, berberine (1–2 μM) significantly decreased MMP-13 (Figure 4A), N-cadherin, RhoA and ROCK-1 (Figure 4B), SOS-1, GRB2, Ras, p-ERK1/2 and p-c-Jun (Figure 4C), p-FAK, p-AKT, NF-κB, and uPA (Figure 4D). However, it increased TIMP-1 (Figure 4A), E-cadherin, PKC (Figure 4C), and PI3K (Figure 4D) in A375.S2 cells after 24 h of treatment, but did not significantly affect MMP-1 and MMP-2 (Figure 4A). Based on these findings, berberine may have suppressed the cell metastasis of A375.S2 cells through multiple signaling pathways.

### 2.5. Berberine Decreases Cell Viability of A375.S2/PLX Resistant Cells

**Figure 5:**
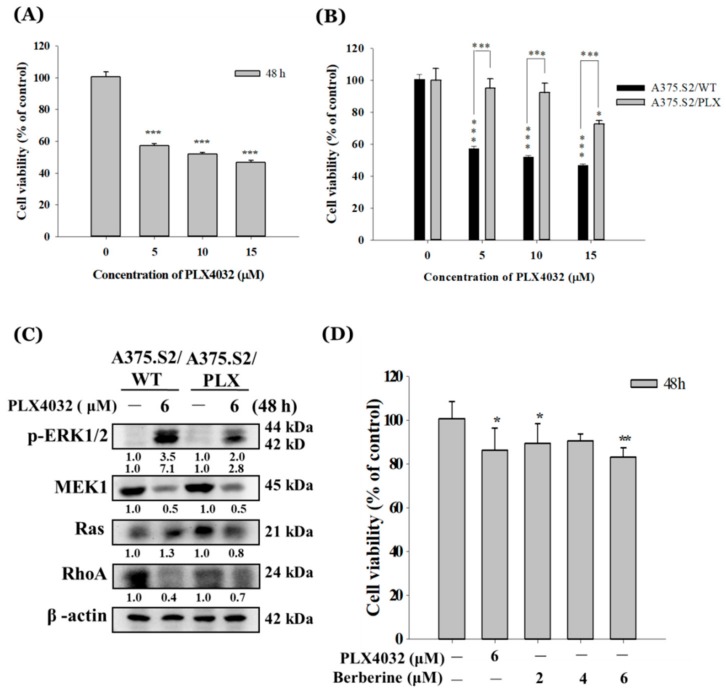
PLX4032 and berberine decreased the viable cell number in A375.S2 cells and PLX4032 resistant A375.S2 cells. The A375.S2 cells were treated with 0, 5, 10, and 15 μM of PLX4032 and were harvested for cell viability (**A**). PLX4032 (6 μM) was used to generate the resistant A375.S2 cells and wild-type A375.S2 cells (A375.S2/WT cells) and the PLX403 resistant A375.S2 cells (A375.S2/PLX cells) were treated with PLX4032 at 0, 5, 10, and 15 μM and were measured for the total viable cell number (**B**). Alternatively, cells were treated with PLX4032 (6 μM) and were harvested for western blotting and protein expression of p-ERK1/2, MEK1, Ras, and RhoA were examined (**C**) as described in the Materials and Methods. PLX4032 resistant A375.S2 cells treated with berberine (0, 2, 4 and 6 μM) for 48 h and were collected for measuring the total viable cell number (**D**) as described in the Materials and Methods. The results were obtained from three independent experiments. * *p* < 0.05, ** *p* < 0.01, *** *p* < 0.001, significant difference between berberine-treated groups and the control as analyzed by one-way ANOVA analysis of variance.

The A375.S2 cells were treated with 0, 5, 10, and 15 μM of PLX4032 (an inhibitor of the BRAF^V600E^ mutation) for 48 h and the cells were collected for measuring the total viable cell numbers. The results are given in Figure 5A. The results indicated that PXL4032 decreased the total viable cell number at 5–15 μM after 48 h of treatment. The IC_50_ was calculated and the value is 6 μM of berberine. The 6 μM concentration of PLX4302 was used to generate the PLX4032 resistant A375.S2 cells (A375.S2/PLX cells). The A375.S2/PLX and A375.S2 (A375.S2/WT) cells were treated with various concentrations of PLX4032 and the results were shown in Figure 5B. The original A375.S2 cells decreased the total viable cells at 5–15 μM of PLX4032 but the A375.S2/PLX cells only reduced the total viable cells with a treatment of 15 μM, but treatments of 5–10 μM did not significantly affect the total viable cells. In order to confirm the PLX4032 resistance of the A375.S2/PLX cells and whether they were involved in the ERK pathway, the western blotting of the associated protein expressions were examined and the results are shown in Figure 5C, which indicates that PLX4032 has a higher inhibition of p-ERK1/2 in A375.S2/PLX cells than in A375.S2/WT cells. PLX4032 inhibited a MEK1 expression in A375.S2/WT and A375.S2/PLX cells. However, PLX4032 inhibited the expression of Ras in A375.S2/PLX cells but increased it in A375.S2/WT cells. Nevertheless, PLX4032 decreased the expression of RhoA in both cells. The A375.S2/PLX cells were treated with PLX4032 (6 μM) and berberine at 2–6 μM for 48 h and the results are shown in Figure 5D, which indicate that berberine reduced the total viable A375.S2/PLX cells at 2–6 μM.

### 2.6. Berberine Suppresses Cell Mobility in A375.S2/PLX Resistant Cells

**Figure 6:**
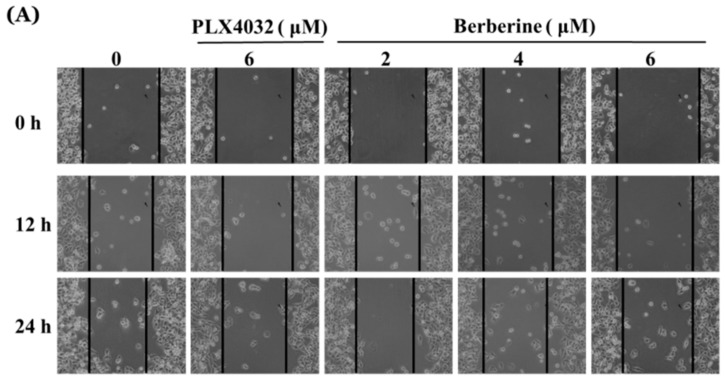

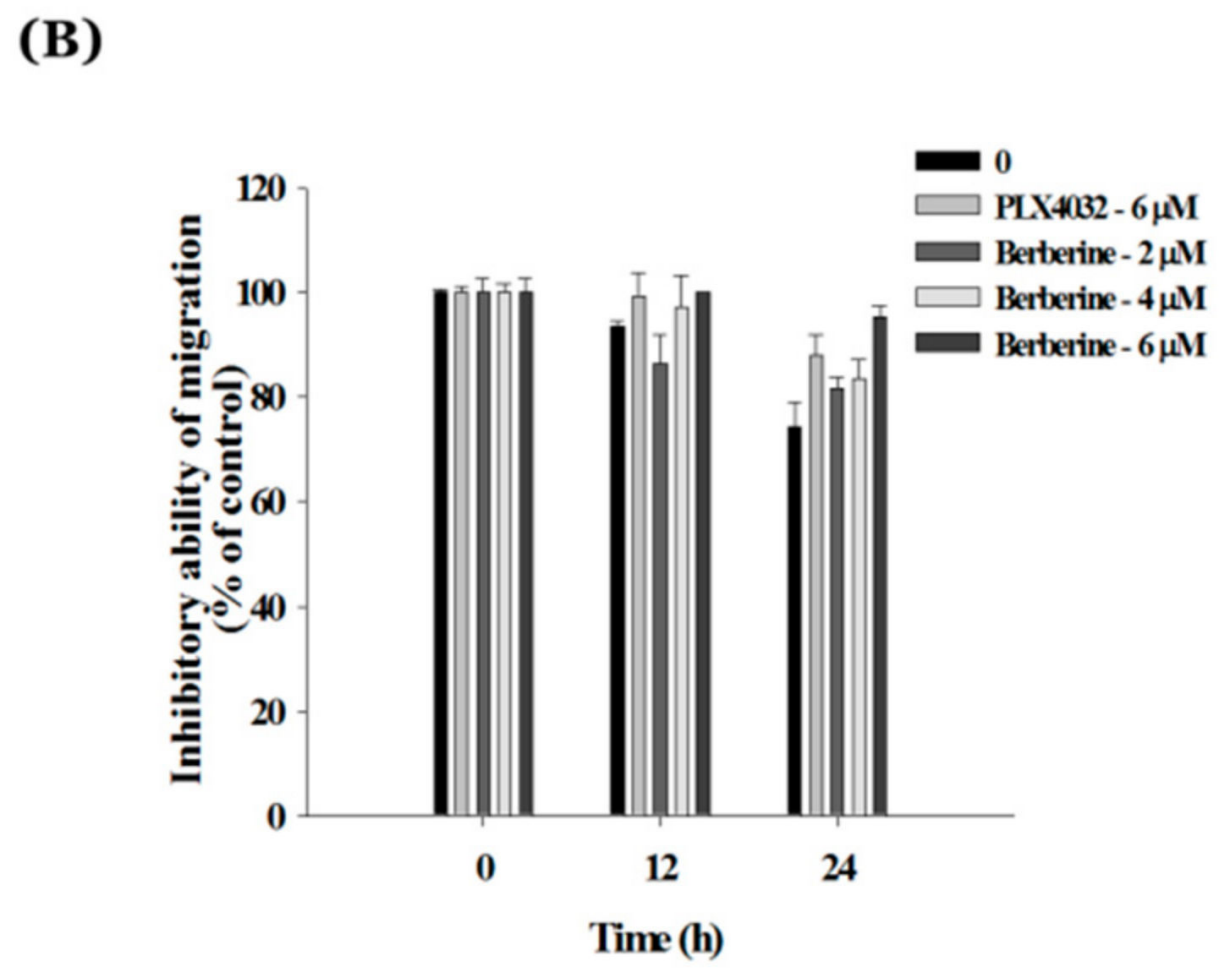
Berberine affected the in vitro wound closure of A375.S2/PLX resistant cells. The cells (2 × 10^5^ cells/well) were kept in 12-well plates for 24 h, were scratched, and were incubated with PLX4032 or berberine (0, 2, 4, and 6 μM) for 48 h. The relative wound closures were photographed using phase contrast microscopy (**A**) and the percentage of inhibitory abilities was calculated (**B**) as described in the Materials and Methods. * *p* < 0.05, significant difference between berberine-treated groups and the control, as analyzed by one-way ANOVA analysis of variance.

As indicated in Figure 6A,B, berberine treatment of 2–6 μM for cell mobility inhibited the scratch area of the wound healing assay and the results indicated that berberine significantly suppressed the cell mobility in a dose-dependent manner. These effects indicated that berberine has a higher inhibition of cell mobility than that of PLX4032 at 6 μM (Figure 6A,B).

## 3. Discussion

It is well documented that chemotherapy drugs contain anti-tumor activities involved in the inhibition of the proliferation, induced cell apoptosis, or suppressed cell metastasis of tumor cells [30,31,32,33]. Numerous pieces of evidence have shown that tumor metastasis may lead to multiple organ failures and dyscrasia, which are the leading causes of death in patients with malignant tumors. Therefore, one of the best strategies against cancer cells is to block the signaling pathway of the cancer cell metastasis [33,34,35]. Many clinical drugs are used for patients with cancer metastasis and some of these drugs are obtained from natural products. Berberine, a compound obtained naturally from plants, has shown to induce cancer cell death in many human cancer cell lines. Moreover, berberine suppresses the migration and invasion of B16F10 murine melanoma cells and A375 human melanoma cells through a reduction in the activity of the ERK signaling pathway and the COX-2 protein levels [36]. However, there is no available information to shows that berberine affects the migration and invasion of A375.S2 and PLX4032-resistant A375.S2 cells. PLX4302 is an inhibitor of the BRAF^V600E^ mutation for the treatment of cancers harboring activated BRAF mutations [37]. Thus, herein, we investigated the effects of berberine cell migration and invasion in A375.S2 cells and PLX4032-resistant A375.S2 cells (A375.S2/PLX) in vitro.

The results indicated that berberine slightly induced cell morphological changes and decreased the viable number of A375.S2 cells at 1–2 μM after 24 h of treatment (Figure 1A,B). Berberine has significantly suppressed the migration of A375.S2 cells in a dose-dependent manner (Figure 2A), however, only at 2 μM after 12 h of treatment does cell mobility get inhibited (Figure 2B). This is in agreement with other reports that showed that berberine suppresses cell mobility in murine melanoma B16 cells [38] and in hepatocellular carcinoma cells [39]. Berberine inhibits the invasion of human skin squamous cell carcinoma A431 cells [29]. 

For further investigation, the results of the cell migration and invasion assay indicated that berberine suppressed the cell migration at 1.5–2 μM after 24 h of treatment (Figure 3B) and inhibited the cell invasion at 1–2 μM after 24 h of treatment (Figure 3C) in A375.S2 cells. These findings are in agreement with other reports that indicated that berberine suppressed the migration and invasion of murine melanoma B16 cells [38], human colorectal cancer SW620 and LoVo cells [28], and human prostate cancer cells in vitro [27]. Gelatin zymography was used to measure the MMP-2 and MMP-9 gelatin activities and the results show that at 2 μM of berberine, MMP-9 activity is significantly reduced after 24 h of treatment. Meanwhile, other investigators have shown that the MMPs activities were markedly suppressed by berberine in a dose-dependent manner in murine melanoma B16 cells [38]. Furthermore, the agent-inhibited MMP-2 could lead to the suppression of tumor metastasis [40,41].

The proteins involved in migration and invasion were investigated by western blotting and the results are shown in Figure 4A–D. As shown in Figure 4B, berberine increased E-cadherin and decreased N-cadherin in A375.S2 cells. It is well known that cancer cell migration and invasion will result in decreased E-cadherin and increased N-cadherin rates [42,43], which play an important role in cancer cell migration and invasion [44,45]. Thus, berberine increased E-cadherin rate and berberine decreased N-cadherin rate (Figure 4B) may be involved in the inhibition of the migration and invasion of A375.S2 cells. Berberine decreased the RhoA (Figure 4B), p-FAK, and p-AKT (Figure 4D) protein expressions at 1–2 μM after berberine treatment for 24 h in A375.S2 cells. The activated PI3K/AKT signaling has shown to involve tumor cell invasion and oncogenesis [45,46], including melanoma cells [47]. Numerous pieces of evidence have shown that RhoA plays a critical role in cell metastasis [48,49,50]. Akt and focal adhesion kinase (FAK) play important roles in glioma and prostate cell migration and invasion [51,52]. The results also showed that berberine (1–2 μM) significantly inhibited the protein expressions, such as SOS-1, GRB2, Ras, p-ERK1/2, p-c-Jun (Figure 4C), NF-κB, and uPA (Figure 4D) in A375.S2 cells. GRB2, SOS-1, Ras, p-ERK1/2, and p-c-Jun have been shown also to involve cell metastasis [53,54]. However, berberine increased the expression of PKC (Figure 4C) and PI3K (Figure 4D) in A375.S2 cells. Thus, further investigations are needed in the future.

More interesting is that the inhibition of the PI3k/Akt pathway led to the decrease in the invasion of melanoma cells [48,55]. the results from Figure 4D also showed that berberine suppressed the expression of p-AKT in A375.S2 cells. The PI3K/Akt pathway plays a role in the MMPs for uPA gene regulation, cell survival, and cell invasion [56,57]. The AKT activation induced the invasion and metastasis of cancer cells by stimulating secretions of MMPs [48,53]. Figure 4D also shows that berberine inhibited the uPA and NF-κB protein expressions in A375.S2 cells. It was reported that the down-regulation of uPA by berberine decreased the HCC cell invasion and migration [39]. MMPs were up-regulation by uPA and tPA and down-regulated by TIMPs and PAI-1 [39,58]. NF-κB was linked with tumor cell proliferation, survival, invasion, and metastasis [59]. Based on these results, we suggest that the berberine-inhibited cell migration, and invasion are involved with NF-κB in A375.S2 cells in vitro.

PLX4032, also known as vemurafenib, is an inhibitor that binds to the ATP-binding site of mutated BRAF kinase, inhibiting ERK signaling only in tumor cells expressing BRAF^V600E^ mutations [60]. The cells develop a resistance to PLX4032 usually within one-year of therapy [61]. Therefore, we generated PLX4032 (inhibitor of the BRAF^V600E^ mutation) resistant A375.S2 cells (A375.S2/PLX cells), which are shown in Figure 5A,B. The A375.S2/PLX cells were treated with berberine at 2, 4, and 6 μM, leading to the significantly reduced total cell number. Furthermore, we also investigated the cell mobility by using the wound healing assay and the results showed that berberine significantly suppressed the cell mobility of the A375.S2/PLX cells (Figure 6A,B). Based on these results, berberine suppressed the cell migration in A375.S2/PLX cells.

In conclusion, our results indicated that berberine significantly suppressed the mobility, migration, and invasion of A375.S2 cells involved in the inhibition of metastasis-associated proteins such as FAK, RhoA, ROCK1, or p-AKT, NF-κB, and uPA, which lead to the inhibition of MMP-1 and MMP-13 in vitro. Overall, the possible signal pathway for berberine-suppressed cell mobility, migration, and invasion of A375.S2 cells and by the FAK, uPA and NF-κB signaling pathways. Furthermore, we also found that berberine suppressed the mobility of PLX4032 resistant A375.S2 cells (A375.S2/PLX cells). Berberine can be considered to have potential as a chemotherapeutic agent in melanoma, which will have to be proven in further in-depth studies, including investigations on in vivo efficacy.

## 4. Materials and Methods

### 4.1. Test Chemicals, Reagents and Culture Medium

Berberine, dimethyl sulfoxide (DMSO), Tris-HCl, trypan blue, trypsin, propidium iodide (PI), gelatin, Coomassie blue R-250, and PLX4032 were purchased from Sigma Chemical Co. (St. Louis, MO, USA). Fetal bovine serum (FBS), Minimum Essential Medium (MEM) culture medium, and penicillin-streptomycin were purchased from Invitrogen (Carlsbad, CA, USA). Primary antibody anti-MMP-1, -MMP-2, and -MMP-13 were obtained from Santa Cruz Biotechnology (Santa Cruz, CA, USA) and anti-TIMP-1, -E-cadherin, -N-cadherin, -RhoA, -ROCK-1, -SOS-1, -GRB2, -Ras, -p-ERK1/2, -p-c-Jun, -PKC, -p-FAK, -PI3K, -p-AKT, NF-κB, -uPA, and the peroxidase conjugated secondary antibodies were purchased from Cell Signaling Technology, Inc. (Beverly, MA, USA). Berberine was dissolved in DMSO as a carrier solvent and control cultures were 0.5% DMSO. Berberine was further diluted in a culture medium to the appropriate final concentrations prior to use.

### 4.2. Cell Line and Culture

The human melanoma A375.S2 cell line with a BRAF^V600E^ mutation was obtained from the Food Industry Research and Development Institute (Hsinchu, Taiwan). The A375.S2 cells were cultured in the MEM medium supplemented with 10% FBS, 2 mM L-glutamine, 10 g/L non-essential amino acid, 100 μg/mL streptomycin, and 100 units/mL penicillin in a humidified atmosphere of 5% CO_2_ at 37 °C and at 70% confluence. The cells displayed a normal morphology as previously described [62,63].

### 4.3. Cell Morphological Examination and Viability Assay

The A375.S2 cells (1 × 10^5^ cells/well) were seeded onto 12-well plates overnight with a MEM culture medium and they were incubated with berberine at final concentrations (0, 1, 1.5, and 2 μM) in triplicate for 24 h. After treatment, the cells were examined and photographed under contrast-phase microscopy at 200×. The cells were harvested, washed with PBS, and were stained with PI (5 μg/mL) for measuring the total percentage of cell viability by using flow cytometry (Becton-Dickinson, San Jose, CA, USA) as described previously [62,63].

The A375.S2 cells (1 × 10^5^ cells/well) were treated with various concentrations (5, 10, or 15 μM) of PLX4032, an inhibitor of the BRAF^V600E^ mutation, for 48 h and the cells were harvested to measure the total viable cell number as described previously [62,63]. To calculate the IC_50_ of the PLX4032, 6 μM of A375.S2 cells were used to generate the PLX4032-resistant A375.S2 cells, as described previously with modifications [64]. The resistant A375.S2 cells were incubated with various concentrations (0, 5, 10, or 15 μM) of PLX4032 for 48 h to measure the total viable cell number. The resistant A375.S2 cells were treated with various concentrations (2, 4, and 6 μM) of berberine for 48 h and were harvested for measuring the total viable cell number as described previously [62,63].

### 4.4. In-Vitro Scratch Wound Healing Assay

Cell mobility characteristics were analyzed by a wound healing assay, as described previously [62,63]. Briefly, A375.S2 or A375.S2/PLX cells (2 × 10^5^ cells/well) were placed in 12-well plates for 24 h and grown until reaching a confluent monolayer. The culture media were replaced with serum-free MEM to wash the cell monolayers. Cell monolayers were scratched (wound) using a sterile 200 μL-pipette tip and to remove the cell debris by PBS washing. A375.S2 or A375.S2/PLX were incubated with various concentrations of berberine (0, 1, 1.5, and 2 μM, or 0, 2, 4, 6 μM, respectively) for different time-periods (0, 12, and 24 h). The migrating cells in the denuded zone were monitored and photographed under phase contrast microscopy. The scratch experiments were done 3 times. We quantitated the relative wound size by using the Image J version 1.49o software. The inhibitory ability of migration is as follows: (% of control) = ((wound area_berberine 12 h or 24 h_/wound area_berberine 0 h_)/(wound area_control 12 h or 24 h_/wound area_control 0 h_)) × 100%, as described previously [65,66].

### 4.5. Gelatin Zymography Assay

The A375.S2 cells (2 × 10^5^ cells/well) were maintained in 12-well plates for 24 h and a serum-free MEM medium containing berberine (0, 1, 1.5, and 2 μM) was individually added to each plate and cultured for 12 and 24 h. The conditioned medium was collected and the total proteins from each treatment were quantitated to load them onto 10% polyacrylamide gels. They were then copolymerized with 0.2% gelatin and then the gel was incubated in a zymogen developing buffer (Sigma-Aldrich, St. Louis, Missouri, USA) containing 50 mM Tris (pH 7.5), 200 mM NaCl, 5 mM CaCl_2_, 1 μM ZnCl_2_, and 0.02% Brij-35. The gel was kept overnight at 37 °C and soaked twice in 2.5% Triton X-100 in dH_2_O at 25 °C for 30 min. The bands of MMP-2 and -9 corresponded to the activity, which was stained with 0.2% Coomassie blue in 10% acetic acid and 50% methanol. After staining, the gel was photographed and the band of gelatinolytic activity was determined using the NIH Image J software, version 1.47 (National Institutes of Health, Bethesda, MA, USA), as described previously [65,67].

### 4.6. Transwell Assay for Cell Migration and Invasion Examinations

Collagens and the Matrigel cell migration and invasion assay system were used to measure the migration and invasion of cell in vitro, as described previously [63,65]. For the cell migration assay, A375.S2 cells (5 × 10^4^ cells/well) in serum-free MEM with berberine (0, 1, 1.5 and 2 μM) were placed in the upper chamber (8 μm pore size; Millipore, Temecula, CA, USA) and coated with 50 μL collagen in each transwell insert overnight, and 800 μL of MEM with 10% FBS was placed in the lower chamber and incubated for 24 h. After incubation, the cells adhering to the upper surface of the membrane were removed and all the migrated cells which adhered to the lower surface were fixed with 4% formaldehyde in PBS, treated with methanol, and stained with 2% crystal violet. After staining, all the samples were examined and photographed under light microscopy to count the total cells and calculate the percentage of inhibition based on the cells on each picture, as described previously [63,65]. The invasion assay was performed in almost the same way with a cell migration assay, except matrigel (matrigel:serum-free medium 1:10) was on the transwell membrane, as described previously [63,65].

### 4.7. Western Blotting Analysis for Cell Metastasis-Associated Protein Expressions

The A375.S2 cells were placed in 10-cm culture dishes at a density of 1 × 10^6^ cells/dish and they were incubated with berberine (0, 1, 1.5, and 2 μM) for 24 h. After treatment, the cells were harvested and re-suspended in a lysis buffer of 50 mM, Tris-HCl pH 7.5, 400 mM NaCl, 2 mM EGTA, 1 mM EDTA, 1 mM DTT, and a protease inhibitor cocktail (Roche, Mannheim, Germany). The cell lysates were centrifuged at 10,000× *g* at 4 °C for 10 min. We quantitated the total protein concentration of the supernatants by using a Bradford protein assay kit. A total of 30 μg of total proteins were separated by 12% SDS-polyacrylamide gel electrophoresis. They were then transferred onto a PVDF membrane (Millipore, Bedford, MA, USA). The membrane was blocked with 5% non-fat milk which was in the TBS-T buffer (10 mM Tris–HCl, 150 mM NaCl, and 0.05% Tween-20, pH 7.8) for 1 h at room temperature. The membranes were washed with the TBS-T buffer and were incubated with monoclonal antibodies such as anti-MMP-1, -MMP-2, -MMP-13, -TIMP-1, -E-cadherin, -N-cadherin, -RhoA, -ROCK1, -SOS-1, -GRB2, -Ras, -p-ERK1/2, p-c-Jung, -PKC, -p-FAK, -PI3K, -p-AKT, -NF-κB, and -uPA. After washing, the membranes were incubated with diluted corresponding HRP-conjugated secondary antibodies (diluted 1:5000; Santa Cruz Biotechnology), developed with ECL (Amersham, Piscataway, NJ), and they were detected directly with a Biospectrum Imaging System (UVP, Inc., Upland, CA, USA) [65,68].

After the PLX4032 treatment, the A375.S2 and PLX4032-resistant A375.S2 cells were harvested for examining the cell migration and invasion expression associated proteins such as p-ERK1/2, MEK1, Ras, and RhoA, as described in the western blotting section.

### 4.8. Statistical Analysis

The results are presented as mean ± SD. The data were statistically analyzed with one-way ANOVA analysis of variance. * *p* < 0.05, ** *p* < 0.01, *** *p* < 0.001 are determined as significant between the control and berberine treated groups.
